# Battling quiescence for tumor eradication: too good to be true?

**DOI:** 10.18632/oncotarget.26452

**Published:** 2018-12-18

**Authors:** Nami McCarty

**Affiliations:** Nami McCarty: Brown Foundation Institute of Molecular Medicine for the Prevention of Human Diseases (IMM), University of Texas-Health Science Center at Houston, Houston, TX, USA

**Keywords:** quiescence, cancer, TRIM, autophagy, deubiquitinase

Efforts to differentiate mechanisms underlying tumor quiescence and proliferation have occurred mostly in leukemia [1], and in hormone-driven cancers of the breast [2] or the prostate [3, 4]. In our previous study, we isolated quiescent cells in multiple myeloma (MM) using a lipophilic dye PKH. We allowed the cells to lose PKH intensity *in vivo* rather than *in vitro* to effectively track the location of quiescent stem-like cells [5]. These cells preferentially reside within the osteoblastic (OS) niche rather than in the vascular (VS) niche of the BM or spleen. Even though some quiescent PKH^+^ cells were found in spleens, these cells were not be able to form the colonies similar to proliferative PKH-CD138^+^ (human plasma B cell marker) cells. In addition, these quiescent cells were tumorigenic upon serial transplantation and were resistant to a variety of clinically relevant chemotherapeutic drugs. Our study is the first to demonstrate a quiescent MM cell niche and the effects of functional interactions between quiescent MM cells and their microenvironment [5].

Molecular profiling of these cells revealed a relatively unknown protein, tripartite motif (TRIM) containing 44, TRIM44, which is highly expressed in quiescent PKH^+^ MM cells isolated from the OS niche compared to proliferating PKH-CD138^+^ MM cells (CD138 is a plasma cell marker) or PKH^+^ MM cells from other niches. A search of the integrated cancer microarray database revealed that TRIM44 mRNA expression is significantly upregulated in MM compared to normal or MGUS (a precursor stage of MM) [6, 7]. This indicates that TRIM44 expression may be also correlated to MM progression. Complete TRIM44 silencing by CRISPR-CAS9 led to MM cell death, suggsting importance of TRIM44 in MM cell survival. One mechanism of TRIM44-mediated tumor cell growth is via deubiquitination of HIF-1α, which is a key hypoxia-induced transcription factor that regulates MM tumor growth, angiogenesis and bone destruction. TRIM44 expression in MM cells also enhances bone destruction in xenograft mice [8]. Therefore, TRIM44 may drive cancer cell survival by managing stress response and/or by stabilizing oncoproteins via deubiquitination.

What are the implications of this finding? Dysregulation of protein ubiquitination and deubiquitination pathways is one of the pathological features of MM [9]. MM cells, which is a malignant form of antibody-secreting plasma cells, constitutively suffer increased ER stress and hence are sensitive to compounds targeting protein homeostasis such as proteasome inhibitors. Bortezomib is one example of how targeting the ubiquitin-proteasome pathway suppresses MM growth [10]. In MM cells, proteasome-associated deubiquitinating enzymes play a central role in modulating the unfolded protein response. Recent studies show that inhibitors targeting enzymes that modulate protein ubiquitination/deubiquitination upstream of the proteasome inhibit MM tumor growth and overcome bortezomib resistance [11]. Since the up-regulation of the deubiquitinase TRIM44 in quiescent MM cells supports their growth in the BM niche and drug resistance, the development of an inhibitor targeting TRIM44 could benefit to targeting quiescent MM cells in the niche and also provide a synergistic therapy to improve the outcome of MM patients.

The TRIM family represents a large family of pattern recognition receptor, and select TRIM proteins have roles as regulators of autophagy as well as cargo receptors [12]. Out of ∼80 human TRIM genes, knock down of 21 TRIMs, including TRIM44, decreased autophagy induction to an extent comparable to or exceeding the effect of Beclin 1 knock down [12]. Even though there are many reports about roles for autophagy in cancer, only recently are there links between autophagy, metabolism, and cancer stem cells. For example, leukemia stem cells exit quiescence leading to an expansion of myeloid progenitors after lysosomal inhibition, which inhibits autolysosomes. Therefore, expression of TRIM44 in quiescent MM cells may be a novel link between autophagy, stem cells and cancer progression.

Furthermore, our work provides a framework to further dissect mechanisms of quiescence and proliferation within the tumor microenvironment. Are particular cells within the microenvironment predisposed to tumor dormancy? What cues trigger tumor cells to exit their quiescent state and become more proliferative? What factors in the microenvironment are involved in maintaining tumor quiescence? These questions are still unanswered but further understanding how tumors remain in quiescence, which allows them to escape effects of chemotherapies, will likely provide a valuable tool to prevent cancer relapse in patients.
